# Supplementary material for: Optimizing the P balance: How do modern maize hybrids react to different starter fertilizers?
Source: PLoS One. 2021 Apr 22;16(4):e0250496. doi: 10.1371/journal.pone.0250496 (PMC8062099; doi:10.1371/journal.pone.0250496)
Supplement: S1 Table — Information of all 20 hybrids investigated in the field season 2019, including the breeding company, maturity (FAO groups go from early 170–220 to late 300–350), the main utilization (’B’ denoting biogas, ’CCM’ corn-cob-mix, ’G’ grain and ’S’ silage), and the companies standard seed treatment. The year of registration is given according to the federal plant variety office. (PDF) [file pone.0250496.s001.pdf]

S1 TABLE. Detailed description of hybrid varieties. Information of all 20 hybrids investigated in the field season 2019, including the breeding company, maturity (FAO groups go from early 170-220 to late 300-350), the main utilization ('B' denoting biogas, 'CCM' corn-cob-mix, 'G' grain and 'S' silage), and the companies standard seed treatment. The year of registration is given according to the federal plant variety office.

Variety	Company	Year of registration	Maturity	Main utilization	Treatment
AGROPOLIS	Agro Mais	2015	S240	S/B/CCM	Mesurol + TMTD
AMAROC	Agro Mais	2016	S230	S/B/CCM	Mesurol + TMTD
AMAVERITAS	Agro Mais	2017	S240/K240	S/G/B	Mesurol + TMTD
BENEDICTIO KWS	KWS	2016	S230/K230	S/G	Mesurol + TMTD
BERNARDINIO KWS	KWS	2018	S240/K240	S/G	Mesurol + TMTD
ES METRONOM	Euralis	2014	S240/K240	S/G/B	Mesurol + Maxim XL
GEOXX	RAGT	2010	S240/K240	S/G/B	Mesurol + Maxim XL + Biofortifier (Geoxx Gold)
HULK	agaSAAT GmbH	2013	S250-260/K250-260	S/G/B/CCM	Maisprotektor (Fungizid + Vogelfraß)
KWS FIGARO	KWS	2016	S250/K250	S/G	Mesurol + TMTD
KWS STABIL	KWS	2013	S200/K200	S/G	Mesurol + TMTD
LG 30.222	Limagrain	2010	S210/K220	S/G	Mesurol + Maxim XL
LG 30.258	Limagrain	2016	S240/K240	S/G/B	Mesurol + Maxim XL
LG 32.16	Limagrain	2007	S260/K240	S/G/B	Mesurol + Maxim XL
P 8666	Pioneer	2017	S260/K250	S/G/B	Mesurol + Maxim XL
P 8329	Pioneer	2016	S250/K240	G	Mesurol + Maxim XL
RICARDINIO	KWS	2008	S230/K220	S/G	Mesurol + TMTD
SIMPATICO KWS	KWS	2014	S250/K260	S/G	Mesurol + TMTD
SY KARDONA	Syngenta	2014	S250/K240	S/G	Mesurol + Maxim XL
SY TALISMAN	Syngenta	2015	S220/K230	S/G	Mesurol + Maxim XL
WALTERINIO KWS	KWS	2015	S270/K270	S/G	Mesurol + TMTD
