# Supplementary material for: Optimizing the P balance: How do modern maize hybrids react to different starter fertilizers?
Source: PLoS One. 2021 Apr 22;16(4):e0250496. doi: 10.1371/journal.pone.0250496 (PMC8062099; doi:10.1371/journal.pone.0250496)
Supplement: S2 Table — Control (Co), triple superphosphate (TSP), calcium ammonium nitrate (CAN), diammonium phosphate (DAP). In brackets the nitrogen and phosphorus content are given in percent. (PDF) [file pone.0250496.s002.pdf]

S2 TABLE. **Starter fertilizer-location combinations.** Control (Co), triple superphosphate (TSP), calcium ammonium nitrate (CAN), diammonium phosphate (DAP). In brackets the nitrogen and phosphorus content are given in percent.

**Starter fertilizers (N / P)[%]**	**Co (- / -)**	**TSP (- / 20))**	**CAN (26 / -)**	**DAP (18 / 20)**
Hohenheim	0 kg/ha	115 kg/ha	77 kg/ha	115 kg/ha
Eckartsweier	0 kg/ha	115 kg/ha	-	-
Dettingen	0 kg/ha	115 kg/ha	-	-
Einbeck	0 kg/ha	-	-	100 kg/ha
Saerbeck	0 kg/ha	-	-	116 kg/ha
