# Supplementary material for: Optimizing the P balance: How do modern maize hybrids react to different starter fertilizers?
Source: PLoS One. 2021 Apr 22;16(4):e0250496. doi: 10.1371/journal.pone.0250496 (PMC8062099; doi:10.1371/journal.pone.0250496)
Supplement: S3 Table — Given for the field season 2019 in each location. (PDF) [file pone.0250496.s003.pdf]

S3 TABLE. Maize cultivation parameters. Given for the field season 2019 in each location.

**Parameters**<br><br>**[Unit]**	**Sowing**<br><br>**[Date]**	**Sowing Depth**<br><br>**[cm]**	**Sowing Density**<br><br>**[plants/m^2]**	**Plot Size**<br><br>**[m^2]**	**Harvest**<br><br>**[Date]**
Hohenheim	April 29th	6	8.8	7.5	October 15th&16th
Eckartsweier	April 23th	4.5	8.8	7.5	September 24th
Dettingen	May 6th	5	8.8	7.5	October 29th
Einbeck	May 1st	5.5	10	18	October 17th
Saerbeck	April 27th	5	10	15.6	October 10th
