# Supplementary material for: Optimizing the P balance: How do modern maize hybrids react to different starter fertilizers?
Source: PLoS One. 2021 Apr 22;16(4):e0250496. doi: 10.1371/journal.pone.0250496 (PMC8062099; doi:10.1371/journal.pone.0250496)
Supplement: S4 Table — Same methods were applied for all locations. (PDF) [file pone.0250496.s004.pdf]

S4 TABLE. **Detailed description of trait assessments.** Same methods were applied for all locations.

Trait	Abbreviation	Description	Unit [ ]
Plant height	PH	The average height of three representative individuals from the ground to the straigthened youngest leave (V-stages) and up to tassel tip for the final measurement (R-stage), respectively	cm
Ear height	EH	The average height of three representative individuals from the ground to the internode of the top (uppermost) ear	cm
Days to anthesis	DTA	Male flowering: 50% pollen shedding in one plot	days after sowing
Days to silking	DTS	Female flowering: 50% silks visible in one plot	days after sowing
Anthesis-silking-interval	ASI	Days between male and female flowering	days
Grain dry matter content	GDM	Percentage of dry matter after 72h at 110°C in relation to fresh weight at harvest	%
Grain yield	GY	Weight of all threshed grain per plot, corrected for its water content, recalculated for one hectare	t dry matter/ha
Phosphorus grain concentration	Pconc	The average concentration of phosphorus in the maize kernels, measured by means of X-ray fluorescence; calibration obtained by inductively coupled plasma optical emission spectrometry (ICP-OES)	ppm = mg/kg
Phosphorus grain content	Pcont	Phosphorus concentration * grain yield	kg/ha
