# Supplementary material for: Optimizing the P balance: How do modern maize hybrids react to different starter fertilizers?
Source: PLoS One. 2021 Apr 22;16(4):e0250496. doi: 10.1371/journal.pone.0250496 (PMC8062099; doi:10.1371/journal.pone.0250496)
Supplement: S5 Table — Traits are abbreviated as follows: Plant height <55 days after sowing (DAS)(PH1), Plant height 56–60 DAS (PH2), Plant height 61–65 DAS (PH3), Plant height 66–70 DAS (PH4), Plant height 71–75 DAS (PH5), Plant height >75 DAS (PHfinal), ear height (EH), all measured in cm; days to anthesis (DTA) and days to silking (DTS), indicated in DAS; anthesis-silking-interval (ASI) in days; grain dry matter content (GDM) in percent; grain yield (GY) in tons dry matter/ha; Phosphorus grain concentration (P conc) measured with X-ray fluorescence in mg P/kg dry matter; and Phosphorus grain content (P cont) in kg P/ha. Control (Co, grey), starter fertilizers: triple superphosphate (TSP, purple), calcium ammonium nitrate (CAN, blue), diammonium phosphate (DAP, red). (PDF) [file pone.0250496.s005.pdf]

S5 TABLE. Repeatabilities in the single locations. Traits are abbreviated as follows: Plant height <55 days after sowing (DAS)(PH1), Plant height 56-60 DAS (PH2), Plant height 61-65 DAS (PH3), Plant height 66-70 DAS (PH4), Plant height 71-75 DAS (PH5), Plant height >75 DAS (PHfinal), ear height (EH), all measured in cm; days to anthesis (DTA) and days to silking (DTS), indicated in DAS; anthesis-silking-interval (ASI) in days; grain dry matter content (GDM) in percent; grain yield (GY) in tons dry matter/ha; Phosphorus grain concentration (P conc) measured with X-ray fluorescence in mg P/kg dry matter; and Phosphorus grain content (P cont) in kg P/ha. Control (Co, grey), starter fertilizers: triple superphosphate (TSP, purple), calcium ammonium nitrate (CAN, blue), diammonium phosphate (DAP, red).

	PH1 [cm]	PH2 [cm]	PH3 [cm]	PH4 [cm]	PH5 [cm]	PHfinal [cm]	EH [cm]	DTA [DAS]	DTS [DAS]	ASI [d]	GDM [%]	GY [t DM /ha]	P conc [mg/kg DM]	P cont [kg/ha]
**(i)**	**Hohenheim**													
**Co**	0.85	0.80	0.83			0.71	0.86	0.95	0.96	0.84	0.97	0.62	0.80	0.67
**TSP**	0.64	0.82	0.83			0.78	0.83	0.93	0.95	0.79	0.91	0.49	0.92	0.50
**CAN**	**0.86**	**0.75**	**0.85**			**0.79**	**0.91**	**0.94**	**0.97**	**0.85**	**0.97**	**0.40**	**0.86**	**0.36**
**DAP**	0.65	0.66	0.67			0.91	0.87	0.91	0.95	0.73	0.96	0.62	0.94	0.69
**(ii)**	**Eckartsweier**													
**Co**				0.83		0.67	0.82		0.91		0.94	0.41	0.93	0.40
**TSP**				0.67		0.84	0.81		0.93		0.96	0.66	0.94	0.79
**(iii)**	**Dettingen**													
**Co**	0.51				0.77	0.81	0.82				0.97	0.35	0.92	0.64
**TSP**	0.70				0.90	0.81	0.82				0.97	0.60	0.93	0.66
**(iv)**	**Einbeck**													
**Co**	0.72					0.83	0.92				0.96	0.66		
**DAP**	0.64					0.82	0.97				0.98	0.85		
**(v)**	**Saerbeck**													
**Co**		0.62						0.87	0.90	0.51	0.93	0.36		
**DAP**		0.65						0.92	0.95	0.54	0.98	0.55		
