# Supplementary material for: Optimizing the P balance: How do modern maize hybrids react to different starter fertilizers?
Source: PLoS One. 2021 Apr 22;16(4):e0250496. doi: 10.1371/journal.pone.0250496 (PMC8062099; doi:10.1371/journal.pone.0250496)
Supplement: S6 Table — (i) Control (Co) vs. triple superphosphate (TSP) and (ii) Control (Co) vs. diammonium phosphate (DAP): Values are given for within each starter fertilizer treatment (indicated with Co, TSP, DAP, respectively) as well as across both starter fertilizer treatments. Minimum (Min), Mean, and Maximum (Max) is given based on the best linear unbiased estimators (BLUEs). σ2g denotes the genotypic variance, σ2l the location variance, σ2gxt the genotype-by-treatment-interaction variance, σ2gxtxl the genotype-by-treatment-by-location-interaction variance, σ2e, the error variance, and H2 the broad-sense heritability. Traits are abbreviated as follows: plant height at BBCH stage ~ V4 (PH early), plant height at BBCH stage > R1 (PH late), ear height (EH), days to silking (DTS) given in in days after sowing (DAS), grain dry matter (GDM), grain yield (GY), phosphorus grain concentration (P grain conc), and phosphorus grain content (P cont). Significance levels are shown as ‘*’ (p-value < 0.05), ‘**’ (p-value < 0.01), ‘***’ (p-value < 0.001). All values are based on three locations (Co-TSP: Hohenheim, Eckartsweier, Dettingen; Co-DAP: Hohenheim, Einbeck, Saerbeck), except for P grain conc and P cont in the DAP series. (PDF) [file pone.0250496.s006.pdf]

S6 TABLE. **Summary of the statistical analyses in the series.** (i) Control (Co) vs. triple superphosphate (TSP) and (ii) Control (Co) vs. diammonium phosphate (DAP): Values are given for within each starter fertilizer treatment (indicated with Co, TSP, DAP, respectively) as well as across both starter fertilizer treatments. Minimum (Min), Mean, and Maximum (Max) is given based on the best linear unbiased estimators (BLUEs). σ^2_g denotes the genotypic variance, σ^2_l the location variance, σ^2_{gxt} the genotype-by-treatment-interaction variance, σ^2_{gxtxl} the genotype-by-treatment-by-location-interaction variance, σ^2_e, the error variance, and H^2 the broad-sense heritability. Traits are abbreviated as follows: plant height at BBCH stage ~ V4 (PH early), plant height at BBCH stage > R1 (PH late), ear height (EH), days to silking (DTS) given in in days after sowing (DAS), grain dry matter (GDM), grain yield (GY), phosphorus grain concentration (P grain conc), and phosphorus grain content (P cont). Significance levels are shown as '*' (p-value < 0.05), '**' (p-value < 0.01), '***' (p-value < 0.001). All values are based on three locations (Co-TSP: Hohenheim, Eckartsweier, Dettingen; Co-DAP: Hohenheim, Einbeck, Saerbeck), except for P grain conc and P cont in the DAP series.

	PH early [cm]	PH late [cm]	EH [cm]	DTS [DAS]	GDM [%]	GY [t DM/ha]	P grain conc [mg/kg DM]	P cont [kg P/ha]
				(i) Co vs TSP				
**Min (Co)**	106.67	269.17	115.28	81.25	64.52	11.08	2132	25.13
**Min (TSP)**	108.67	266.94	110.28	80.25	64.73	10.91	2127	25.94
**Mean (Co)**	120.04	296.52	137.56	83.59	69.70	11.75	2362	27.74
**Mean (TSP)**	124.56	293.11	131.41	82.69	70.39	11.89	2379	28.34
**Max (Co)**	130.42	315.83	159.17	86.50	74.65	12.78	2742	30.69
**Max (TSP)**	133.75	310.83	151.17	86.25	74.93	12.99	2796	31.32
**σ^2_g (Co)**	40.84***	128.32***	154.99***	2.74***	4.82***	<0.01	21633.27***	535.25
**σ^2_g (TSP)**	47.84***	109.12***	114.18***	2.44***	5.52***	0.14	27897.23***	863.19
**σ^2_{gxl} (Co)**	<0.01	<0.01	<0.01	0.45**	1.13***	0.20*	6564.03**	2048.21**
**σ^2_{gxl} (TSP)**	<0.01	21.14	21.88	0.62***	1.17***	0.24*	6941.92***	2498.12*
**σ^2_e (Co)**	35.68	79.88	51.77	0.46	0.56	0.50	6876.71	3466.93
**σ^2_e (TSP)**	23.59	62.20	57.78	0.37	0.67	0.47	4730.04	3580.09
**H^2 (Co)**	0.87	0.91	0.94	0.92	0.91	0.02	0.87	0.30
**H^2 (TSP)**	0.92	0.86	0.89	0.90	0.92	0.47	0.90	0.38
**σ^2_g**	45.18***	118.06***	117.32***	2.57***	5.15***	0.08	24392.19***	708.67
**σ^2_l**	3.98**	862.02***	103.27***	6.34***	15.24***	0.59***	5674.53***	7303.70***
**σ^2_{gxt}**	<0.01	11.02	<0.01	0.01	<0.01	<0.01	333.71	<0.01
**σ^2_{gxl}**	2.01	11.02	10.86*	0.56***	1.22***	0.28***	6780.18***	2442.89***
**σ^2_{gxtxl}**	<0.01	<0.01	<0.01	<0.01	<0.01	<0.01	<0.01	<0.01
**σ^2_e**	26.54	60.86	48.07	0.39	0.56	0.41	5790.10	3337.99
**H^2**	0.90	0.90	0.91	0.91	0.91	0.34	0.88	0.34

(ii) Co vs DAP								
**Min (Co)**	119.83	285.00	110.00	82.75	63.08	10.50	2107$^{\$}$	25.69$^{\$}$
**Min (DAP)**	132.56	291.67	113.17	80.00	64.03	10.88	2175$^{\$}$	26.13$^{\$}$
**Mean (Co)**	134.93	311.46	135.31	86.19	67.28	11.16	2383$^{\$}$	29.66$^{\$}$
**Mean (DAP)**	146.46	313.90	135.10	83.84	68.12	11.56	2429$^{\$}$	30.74$^{\$}$
**Max (Co)**	144.22	336.67	162.92	89.25	72.51	12.00	2706$^{\$}$	35.71$^{\$}$
**Max (DAP)**	154.56	334.58	165.00	87.75	72.96	12.12	2836$^{\$}$	34.96$^{\$}$
**σ^2_g (Co)**	39.09***	140.34***	171.04***	2.85***	4.10***	0.09*	19842.73**$^{\$}$	2211.47*$^{\$}$
**σ^2_g (DAP)**	32.48***	112.83***	149.75***	4.49**	4.48***	0.02	25180.66***$^{\$}$	2559.33*$^{\$}$
**σ^2_{gxl} (Co)**	3.54	<0.01	14.07	0.43**	0.45***	0.09	n.a.	n.a.
**σ^2_{gxl} (DAP)**	8.09	10.49	29.70***	1.52***	0.40***	0.25**	n.a.	n.a.
**σ^2_e (Co)**	26.54	70.18	43.02	0.49	0.45	0.30	10000.86$^{\$}$	2980.52$^{\$}$
**σ^2_e (DAP)**	42.06	38.19	22.36	0.63	0.30	0.27	4152.71$^{\$}$	4903.24$^{\$}$
**H^2 (Co)**	0.87	0.92	0.94	0.93	0.95	0.70	0.80$^{\$}$	0.67$^{\$}$
**H^2 (DAP)**	0.77	0.92	0.92	0.88	0.96	0.09	0.92$^{\$}$	0.50$^{\$}$
**σ^2_g**	32.69***	125.21***	162.28***	3.71***	4.34***	0.07*	n.a.	n.a.
**σ^2_l**	310.30***	711.65***	49.25***	0.58*	0.97***	2.35***	n.a.	n.a.
**σ^2_{gxt}**	2.22	0.90	<0.01	<0.01	<0.01	<0.01	n.a.	n.a.
**σ^2_{gxl}**	8.50**	6.73	20.08***	0.60**	0.26***	0.09*	n.a.	n.a.
**σ^2_{gxtxl}**	<0.01	<0.01	<0.01	0.34**	0.12*	0.07	n.a.	n.a.
**σ^2_e**	32.31	53.10	32.89	0.56	0.38	0.28	n.a.	n.a.
**H^2**	0.78	0.92	0.93	0.93	0.97	0.46	n.a.	n.a.

$^{\$}$: only assessed for the location HOH
