# Supplementary material for: Optimizing the P balance: How do modern maize hybrids react to different starter fertilizers?
Source: PLoS One. 2021 Apr 22;16(4):e0250496. doi: 10.1371/journal.pone.0250496 (PMC8062099; doi:10.1371/journal.pone.0250496)
Supplement: S1 Fig — Daily precipitation rates [mm/d] and mean temperatures [°C] of the year 2019. The dates of plant height measurements during the field season are indicated with dark green arrows, the period of the field season with a light green arrow. Soil temperatures at 2 cm, 20 cm, and 200 cm are shown in brown colors in the plot below. (PDF) [file pone.0250496.s008.pdf]

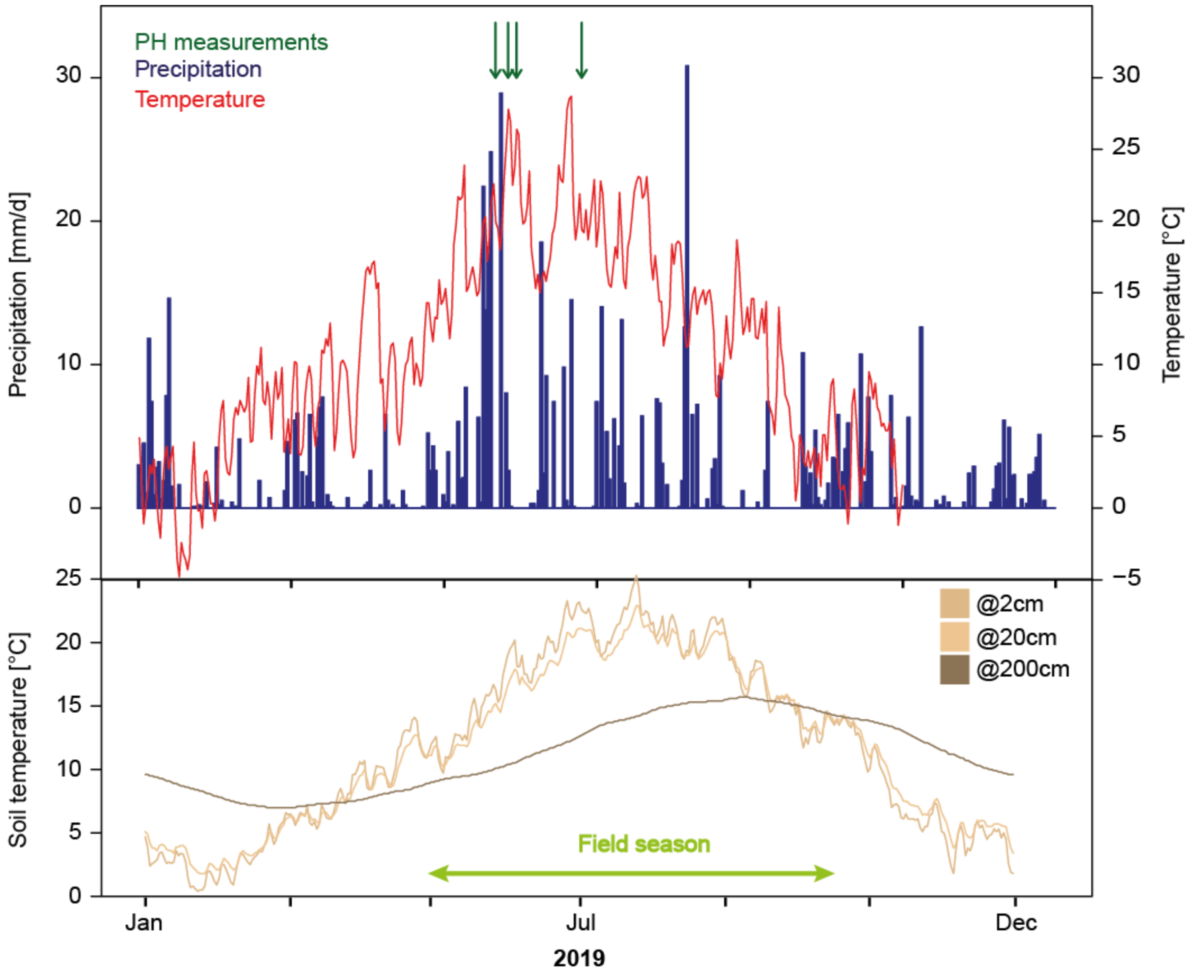

S1 FIG. Climograph and soil temperatures at the location Hohenheim. Daily precipitation rates [mm/d] and mean temperatures [°C] of the year 2019. The dates of plant height measurements during the field season are indicated with dark green arrows, the period of the field season with a light green arrow. Soil temperatures at 2 cm, 20 cm, and 200 cm are shown in brown colors in the plot below.
