# Supplementary material for: Optimizing the P balance: How do modern maize hybrids react to different starter fertilizers?
Source: PLoS One. 2021 Apr 22;16(4):e0250496. doi: 10.1371/journal.pone.0250496 (PMC8062099; doi:10.1371/journal.pone.0250496)
Supplement: S2 Fig — The traits final plant height (PHfinal), ear height (EH), days to anthesis (DTA), days to silking (DTS), and P concentration (Pconc) are depicted. Different letters indicate significant (p-value < 0.05) differences between starter fertilizers means. Starter fertilizers are abbreviated as Control (Co), triple superphosphate (TSP), calcium ammonium nitrate (CAN), diammonium phosphate (DAP). (PDF) [file pone.0250496.s009.pdf]

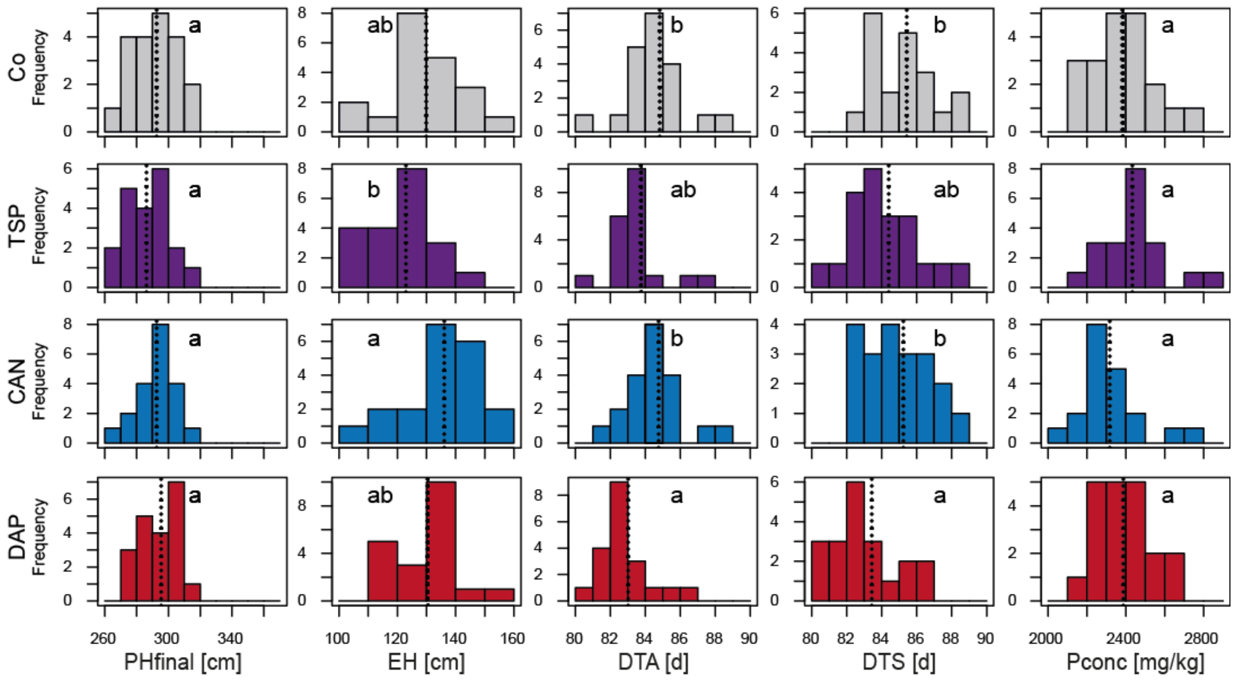

S2 FIG. Histograms of specific traits. The traits final plant height (PHfinal), ear height (EH), days to anthesis (DTA), days to silking (DTS), and P concentration (Pconc) are depicted. Different letters indicate significant (p-value < 0.05) differences between starter fertilizers means. Starter fertilizers are abbreviated as Control (Co), triple superphosphate (TSP), calcium ammonium nitrate (CAN), diammonium phosphate (DAP).
