# Supplementary material for: Optimizing the P balance: How do modern maize hybrids react to different starter fertilizers?
Source: PLoS One. 2021 Apr 22;16(4):e0250496. doi: 10.1371/journal.pone.0250496 (PMC8062099; doi:10.1371/journal.pone.0250496)
Supplement: S3 Fig — Separated by starter fertilizers: control (Co), triple superphosphate (TSP), calcium ammonium nitrate (CAN), diammonium phosphate (DAP). Anthesis-silking-interval (ASI [d]), days to anthesis (DTA [d]), days to silking (DTS [d]), ear height (EH [cm]), grain dry matter content (GDM [%]), grain yield (GY [t/ha]), P grain concentration (Pconc [mg/kg]), P grain content (Pcont [kg/ha]), and plant heights (PH1, PH2, PH3, PHfinal [cm]). Red indicates negative correlations between traits, green positive correlations. Significance levels are shown as ‘.’ (p-value < 0.1), ‘*’ (p-value < 0.05), ‘**’ (p-value < 0.01), ‘***’ (p-value < 0.001). (PDF) [file pone.0250496.s010.pdf]

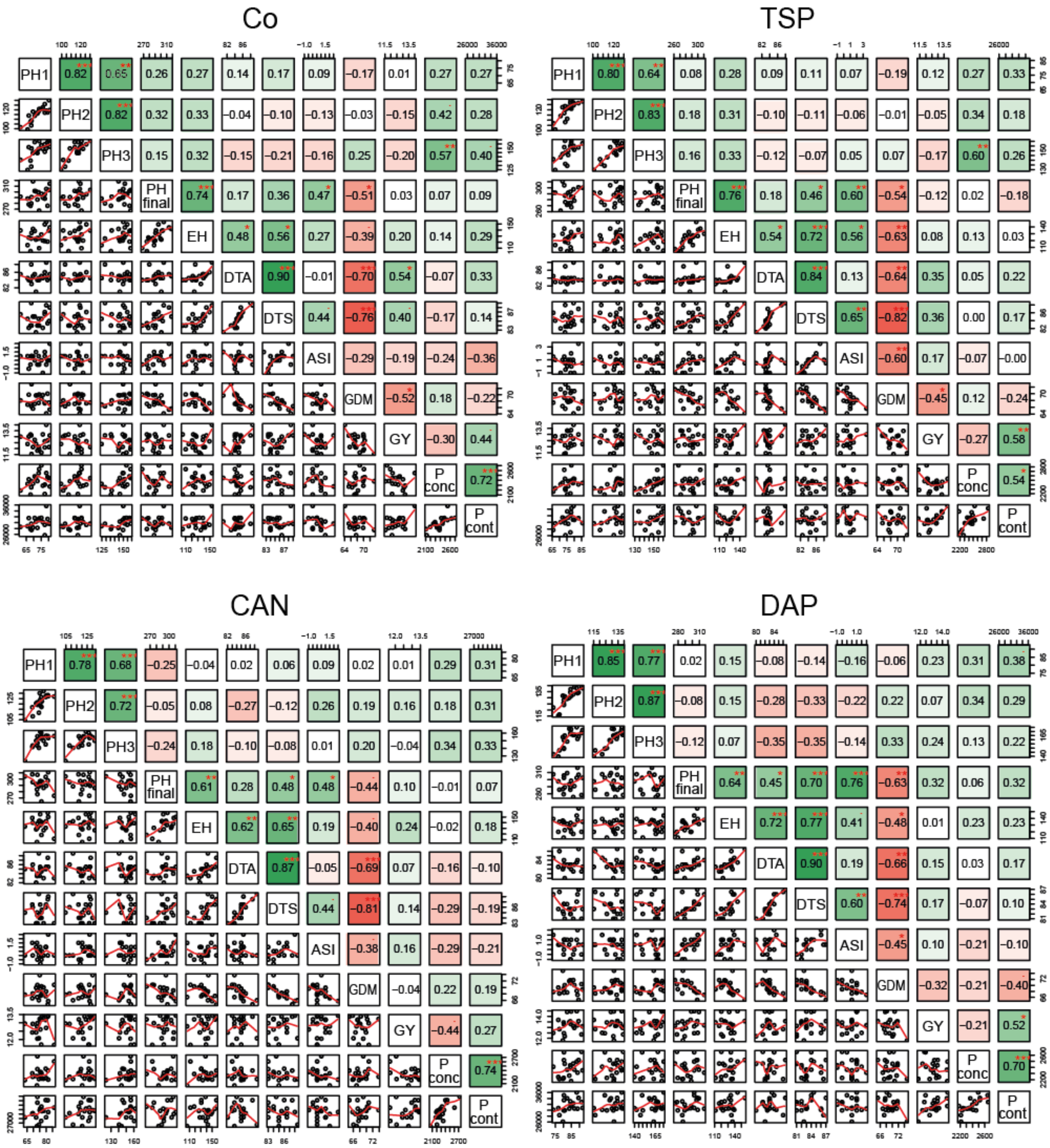

S3 FIG. Correlation matrices of all investigated traits. Separated by starter fertilizers: control (Co), triple superphosphate (TSP), calcium ammonium nitrate (CAN), diammonium phosphate (DAP). Anthesis-silking-interval (ASI [d]), days to anthesis (DTA [d]), days to silking (DTS [d]), ear height (EH [cm]), grain dry matter content (GDM [%]), grain yield (GY [t/ha]), P grain concentration (Pconc [mg/kg]), P grain content (Pcont [kg/ha]), and plant heights (PH1, PH2, PH3, PHfinal [cm]). Red indicates negative correlations between traits, green positive correlations. Significance levels are shown as '.' (p-value < 0.1), '*' (p-value < 0.05), '**' (p-value < 0.01), '***' (p-value < 0.001).
