# Supplementary material for: Optimizing the P balance: How do modern maize hybrids react to different starter fertilizers?
Source: PLoS One. 2021 Apr 22;16(4):e0250496. doi: 10.1371/journal.pone.0250496 (PMC8062099; doi:10.1371/journal.pone.0250496)
Supplement: S4 Fig — 120 grain samples of the core location HOH were analyzed, independent of starter fertilizer treatments. Positive Pearson correlations (r) are indicated in green, negative Pearson correlations in red. (PDF) [file pone.0250496.s011.pdf]

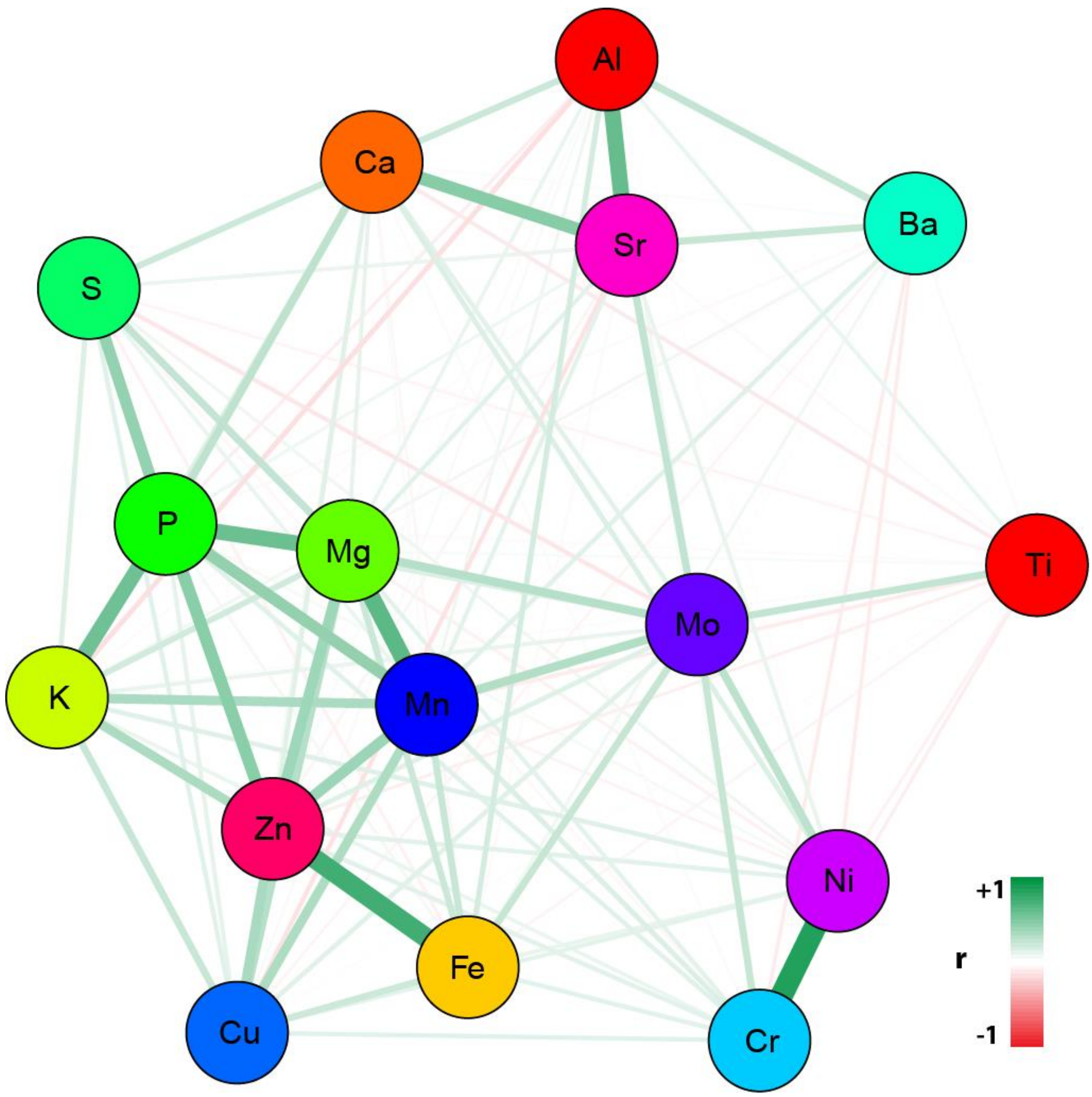

S4 FIG. Network plot among 16 chemical elements. 120 grain samples of the core location HOH were analyzed, independent of starter fertilizer treatments. Positive Pearson correlations (r) are indicated in green, negative Pearson correlations in red.
