# Supplementary material for: Optimizing the P balance: How do modern maize hybrids react to different starter fertilizers?
Source: PLoS One. 2021 Apr 22;16(4):e0250496. doi: 10.1371/journal.pone.0250496 (PMC8062099; doi:10.1371/journal.pone.0250496)
Supplement: S5 Fig — Separated by starter fertilizers (control (Co), triple superphosphate (TSP), calcium ammonium nitrate (CAN), diammonium phosphate (DAP)): anthesis-silking-interval (ASI [d]), days to anthesis (DTA [d]), days to silking (DTS [d]), ear height (EH [cm]), grain dry matter (GDM [%]), grain yield (GY [t/ha]), P concentration (Pconc [mg/kg]), P content (Pcont [kg/ha]), and plant heights (PH1, PH2, PH3, PHfinal [cm]). Dark red indicates maximum, light yellow minimum trait values. (PDF) [file pone.0250496.s012.pdf]

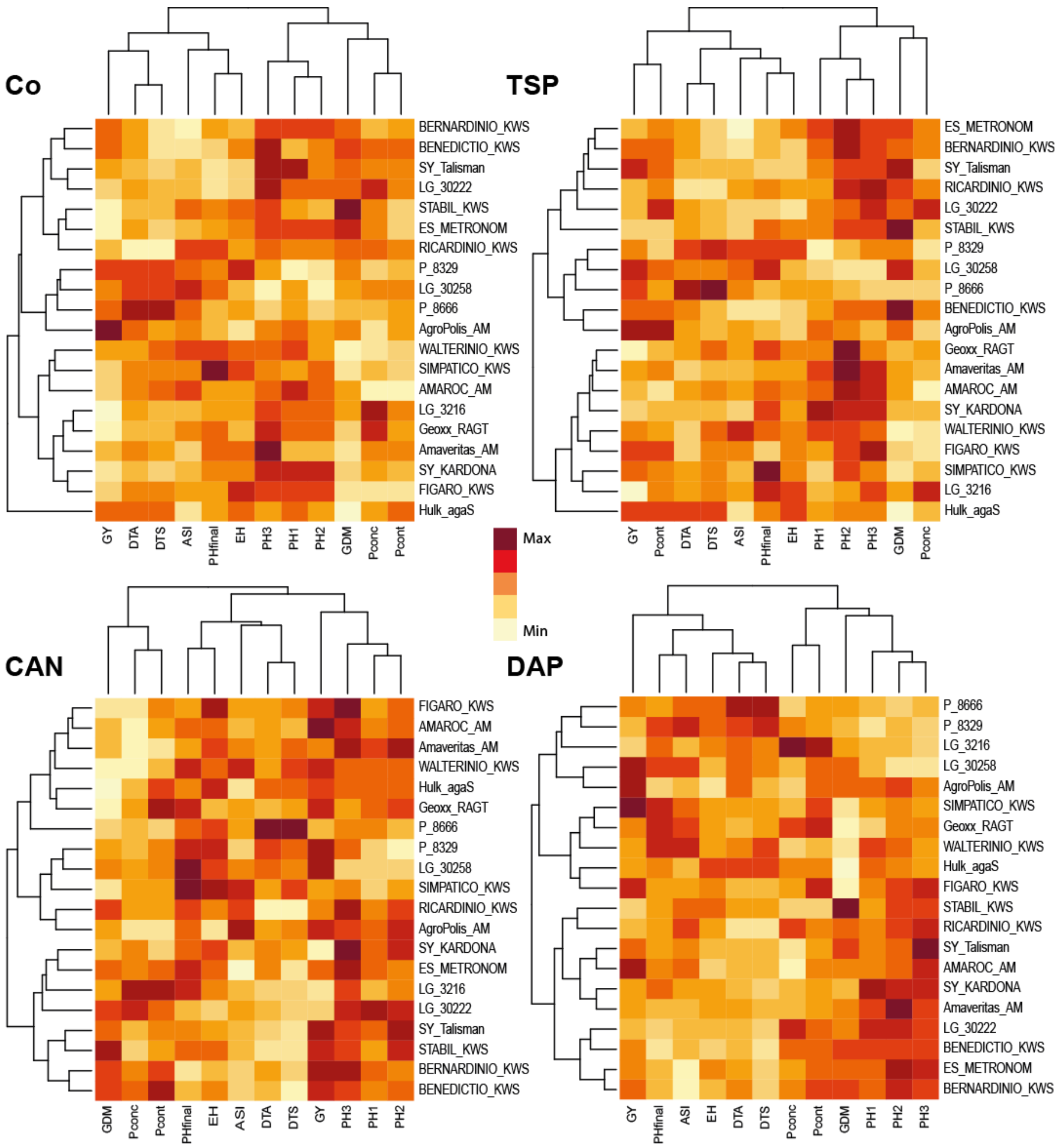

S5 FIG. Heatmaps of all 20 maize hybrids and the investigated traits. Separated by starter fertilizers (control (Co), triple superphosphate (TSP), calcium ammonium nitrate (CAN), diammonium phosphate (DAP)): anthesis-silking-interval (ASI [d]), days to anthesis (DTA [d]), days to silking (DTS [d]), ear height (EH [cm]), grain dry matter (GDM [%]), grain yield (GY [t/ha]), P concentration (Pconc [mg/kg]), P content (Pcont [kg/ha]), and plant heights (PH1, PH2, PH3, PHfinal [cm]). Dark red indicates maximum, light yellow minimum trait values.
