# Supplementary material for: Optimizing the P balance: How do modern maize hybrids react to different starter fertilizers?
Source: PLoS One. 2021 Apr 22;16(4):e0250496. doi: 10.1371/journal.pone.0250496 (PMC8062099; doi:10.1371/journal.pone.0250496)
Supplement: Attachment — Submitted filename: Response to reviewers_revised_PONE-D-20-36545.pdf [file pone.0250496.s015.pdf]

Reviewer #1:

1. In the abstract, authors claimed yield increase of on average +0.67 t/ha (+5.34 %) compared to the control with DAP while in the discussion section they are claiming on average +0.4 t/ha (+3.6 %) higher yield over multiple locations. Please check the same.

Thank you for this helpful hint. While in the abstract, we referred to the core location 'Hohenheim' with a yield increase of +0.67 t/ha, the later mentioned +0.4 t/ha refer to the multi-locational average yield increase across the three DAP locations (also see Table S6).

We hope to eliminate this confusion by adjusting the manuscript accordingly:

Line no. 346 ff.

Our results showed that only the combination of ammonium and phosphate (DAP) as starter fertilization resulted in significantly higher grain yields by on average +0.67 t/ha at the core location Hohenheim (Figs 1b and 3), as well as on average +0.4 t/ha over multiple locations, which corresponds to an increase of +5.34% and +3.6%, respectively (Table S6). By contrast, the yield increase using only phosphate (TSP) as starter fertilizer only amounted to +1.2% across multiple locations.

2. Line no. 73-73:

In many cases however, the abundant P is fixed by minerals and therefore not fully plant available.

May be written as:

In many cases however, the abundant P is fixed by minerals and therefore not fully available for plants.

Done.

3. Line no. 100:

We applied four different starter fertilizers, namely….

Since authors have used three starter fertilizers and one control..So this may be written as:

We applied four treatments i.e. control and three different starter fertilizers…

Done. Additionally, line no. 88 ff. was adjusted in the same manner.

4. Figure S5 and S6 cited before S4 in text. Please re-arrange figures to cite them sequentially.

Thank you very much! The necessary changes have been performed. Figure S4 was numbered S5 and vice versa. However, Figure S6 does not exist. The supplementary Tables S1-S6 were checked for their correct order.

Reviewer #2:
1. Insufficient experimentation: The study involving three fertilizer treatments is conducted at one location and the data is of one year. Other locations do not have all the treatment combinations.

It is correct that we usually use multi-environment trials in breeding in order to estimate the environmental and genetic components contributing to the observed variance – which is what was realized in this study. Please note that we have a sufficient number of three different locations for estimating the starter fertilizer effect of both DAP and TSP. Therefore, we did not realize all starter fertilizer treatments at all locations. The CAN treatment was only added in order to be able to see the effect of ammonium nitrate separately from the effect of phosphate (TSP) in comparison to the combination of ammonium and phosphate (DAP). Based on the data obtained in the field season 2019 we inferred that another year of trials would not give more valuable information to answer the objectives of this study since we have not observed any significant genotype-by-treatment interaction (as shown in Table S6). We hope this additional background information helps to understand what aspects were prioritized and thereby how field trials were set up.

2. The title mentions optimizing P balance and the experiment conducted on P sufficient soils will not give reliable results. Hence, Also G x fertilizer effects cannot be studied from the experimentation and the discussion done on this point is not valid.

We believe that P balance and thus efficient use of this resource is not only of relevance in regions with strongly P-deficient soils. Indeed, compared to other parts of the world, our soils are P sufficient. However, the P problematic in Germany (as well as in many other countries) is a different one that nevertheless requires an optimization of the P balance. Given the fact that we wanted to study P fertilizer applications and their potential reduction in the light of the new fertilizer ordinance, we aimed to perform the experiment under conditions suited to address this question and as realistic and close to practical farming as possible. Consequently, trials took place on well-supplied soils with regard to P. This is the case for the vast majority of actively cultivated fields in Germany (and most other intensively cultivated parts of Europe). Hence, optimizing the P balance has to be understood in the context of reducing P rather than using it as a scarce plant nutrient. The study is valid for Germany and other regions of Europe, where P deficiency is not a current issue. We have emphasized these objectives also clearly in the discussion part (see section '4.2 | Potential for optimizing the P balance on well-supplied soils') and in the conclusions we drew from our results (e.g. line no. 51-53, line no. 397-399).

3. Abstract is not clear

We reread the abstract and changed line no. 49-50 to:
Importantly, the genotype-by-starter fertilizer interaction was always non-significant in the multi-location analysis.

4. ANOVA table not presented. How the experiments analyzed from all locations not given. Also locations are not discussed in material and methods and other sections.

The ANOVA table was presented as Table S6 in the supporting information.
Statistical analyses were described in chapter 2.4 (starting from line no. 146). The full model is given in line no. 155 for the multiple location analysis.
Table 1 (line no. 128 ff.) gives detailed information about the locations, including altitude, weather data (mean temperature, mean precipitation), soil type, pH, and P status.
Moreover, Table S3 provides all cultivation parameters of the field season 2019 recorded for the five different locations.

5. Manuscript has to be improved for clarity in several places and well supported with relevant tables and figures.

We reread the entire manuscript to improve it for clarity. All presented results as well as their discussion are supported by the relevant tables and figures.

6. Authors need to follow standard notations ex. Field seasons, youth etc. used in paper

We hope that the following adjustments are taking the required changes sufficiently into account.
Line no. 125: 'growing period' was replaced by 'field season'.
Line no. 187: 'growing season' was replaced by 'field season'.
Supporting information Table S3: 'growing season' was replaced by 'field season'.
Line no. 184: 'early stage' was replaced by 'youth stage'.
Line no. 185: 'early developmental stages' were replaced by 'youth developmental stages'.
Line no. 333: 'early maize development' was replaced by 'maize youth development'.
Line no. 397: 'early development of maize' was replaced by 'youth development of maize'.

Reviewer #3:

1. The study undertaken was one of the most important aspect which would try to address the issue of development of 'P' use efficient genotypes in the context of 'P' being scarce resource and expected to exhaust in near future. However, based on the following points, the manuscript require major revision in the light of below observations.

2. The authors have used TSP, DAP and CAN to know the difference between different starter fertilizers but authors have not mentioned or studied the effect of other nutrients like nitrogen on 'P' utilization. Because balanced use of fertilizers is advocated for efficient use of fertilizers.

Thank you for highlighting once more the importance of a balanced use of fertilizers (please also see line no. 332 ff.). With regard to the effect other nutrients like nitrogen have on 'P' utilization, we kindly want to refer to section '4.1 | P as starter fertilizer and its interaction with other plant nutrients' (line no. 308 ff.), which is dedicated to this important aspect.
We added the following sentence in line no. 310:
The importance of a balanced nutrient management and specifically the role of nitrogen in fertilization is well known.
Moreover, we investigated the relationship between 16 chemical elements of the maize kernels depending on the starter fertilizer treatment (line no. 231: Figure S4; formerly numbered as Figure S5).

3. Authors are requested to justify the validity of the results in the practical situation of maize cultivation where the soil contains different nutrients in varying amounts, how does the results of the present experiment would apply in practical situation.

In line no. 66 ff. we mentioned the application of over 90.8 Mt of P fertilizers in Germany per annum. Common agricultural practice in maize implies the application of starter fertilizers, mostly DAP. Hence, our study does exactly reflect these farming practices. We understand that nutrients vary in different soils. Therefore, we performed multi-environment trials and analyzed all soils additionally to pH and the P status (Table 1) also for nitrate, magnesium, potassium, and carbon. Nonetheless, for the sake of clarity, we decided to not include these data in the present manuscript.

4. The experimental results are more of an agronomic perspective rather than pure breeding perspective thus the authors are requested to re-orient the results in breeding perspective.

We agree with the reviewer's comment on having included also an agronomic perspective in this study. Hence, we are well aware that this study is at the intersection of plant breeding, crop production and plant nutrition, as we believe that this topic and the results presented in this study are of interest to scientists from different disciplines. Moreover, with the limited number of the 20 economically most important genotypes, a pure breeding perspective did not seem reasonable to us. For these reasons, we consider the manuscript ideally suited for the broader readership and scope of PLOS ONE, as compared to other, more specialized journals of plant breeding.

5. In addition, the following points may be considered while improving the manuscript
a. Youth development is the new terminology used for early development phase it may be clarified by giving leaf stage (like V3/V4/V5/V6) for larger benefit of the readers.

Thank you for this much appreciated hint, which is in line with the comments of reviewer 2. We adjusted the manuscript accordingly.
Since we decided to measure all genotypes for plant height at a certain date (documented in days after sowing, DAS) rather than a certain leaf stage, we did not assess the leaf stage of each genotype for each plant height measurement. Plant height measurements were then categorized over the different locations according to their assessment in DAS. This information is provided in the supporting information, Table S5. Across locations we summarized measurements as PH early at BBCH stage ~ V4 and PH late at BBCH stage > R1. This information is as well given in the supporting information, Table S6.
For the benefit of the readers, we additionally adjusted the following sentences:
Line no. 217 ff:
Independent of the starter fertilizer, the early plant heights PH1, PH2, and PH3 (measured 53, 59, and 63 DAS, respectively) were closely related (0.75 < r < 0.95; p-values <0.01) but are no predictor for the final plant height (measured 94 DAS), nor grain yield.

b. The references namely 1,17,27,28,29 may also be translated into English for the benefit of the readers to know at least the title of the paper.

Done.

c. Sentence in line 73-74 may be revised

Done in accordance with the comment of reviewer 1.

d. Reference 23, does apply to maize please clarify to readers

We adjusted the sentence in line no. 83 ff:

In previous studies, it was shown that the traits early vigor, early-season plant height, flowering, and yield react to P deficiency in sorghum and can thus be considered as P-sensitive traits (23).

e. Reference 24 (line 436 to 438) t is better to use symbol ‘×’ instead of letter ‘x’; is reference 24 relevant, please have a re-look.

We corrected the letter ‘x’ to ‘×’.
Reference 24 is deemed suitable to give an example for previously observed G×E interactions for nutrient use efficiencies.

f. Line 228 may need to be re-looked at for spelling correction of there-of

Corrected.

Reviewer #4:
Comments on MS PONE-D-20-36545 entitled “Optimizing the P balance: How do modern maize hybrids react to different starter fertilizers?”
The study is on optimizing P balance in maize hybrids and how they react to starter fertilizers, the authors have studies how to reduce starter P for maize hybrids. The study has come out with some novel findings on.

Line 61 – correct 1980ies to 1980s

Done.

The introduction is written well, the hypothesis is stated well
The materials section is well written and the experiment is well planned and all the details of the experiment is given in a good way in the section
The results section is written well with all the results obtained given in the section
The discussion section brings out the reasons for the results obtained in a good way
The MS can be accepted after minor revision

Thank you for this positive feedback on our study and the manuscript.
