# Supplementary material for: Optimizing the P balance: How do modern maize hybrids react to different starter fertilizers?
Source: PLoS One. 2021 Apr 22;16(4):e0250496. doi: 10.1371/journal.pone.0250496 (PMC8062099; doi:10.1371/journal.pone.0250496)
Supplement: Attachment — Submitted filename: Reviewers Comments.docx [file pone.0250496.s014.docx]

**Reviewers Comments**

1. Insufficient experimentation: The study involving three fertilizer treatments is conducted at one location and the data is of one year. Other locations do not have all the treatment combinations.
2. The title mentions optimizing P balance and the experiment conducted on P sufficient soils will not give reliable results. Hence, Also G x fertilizer effects cannot be studied from the experimentation and the discussion done on this point is not valid.
3. Abstract is not clear
4. ANOVA table not presented. How the experiments analyzed from all locations not given. Also locations are not discussed in material and methods and other sections.
5. Manuscript has to be improved for clarity in several places and well supported with relevant tables and figures.
6. Authors need to follow standard notations ex. Field seasons, youth etc. used in paper
