# Supplementary material for: Optimizing the P balance: How do modern maize hybrids react to different starter fertilizers?
Source: PLoS One. 2021 Apr 22;16(4):e0250496. doi: 10.1371/journal.pone.0250496 (PMC8062099; doi:10.1371/journal.pone.0250496)
Supplement: Attachment — Submitted filename: Reviewer Comments for Manuscript Number PONE.docx [file pone.0250496.s013.docx]

**Reviewer Comments for Manuscript Number PONE-D-20-36545**

In the current manuscript (PONE-D-20-36545), the authors have investigated the effect of three different starter fertilizers (DAP, TSP, CAN) relative to control on top 20 maize hybrids of Germany at five locations in one season. A number of associated traits were assessed for the same. The study revealed that combination of ammonium and phosphate i.e. DAP as starter fertilization resulted in slight increase of the P content, slightly higher grain dry matter content but significantly higher grain yields over multiple locations. However, there were no statistically significant changes in most of the traits assessed when TSP and CAN applied as starter fertilizers. The genotype-by-starter fertilizer interaction was always found non-significant which indicates that best performing genotypes can be identified irrespective of the starter fertilizer. Further, authors have identified P sensitive and P stable genotypes. At the end authors discuss about How to breed for phosphate-use-efficiency in Germany and advocates that breeders can select P-efficient lines independent of the soil P status since generally the best genotypes performed the best no matter with or without starter fertilization.

I found the manuscript is well written and presented in lucid fashion with sufficient data to support the conclusion. Therefore I can recommend the MS for publication. However my minor concerns to be addressed before final publication/acceptance are:

1. In the abstract, authors claimed yield increase of on average +0.67 t/ha (+5.34 %) compared to the control with DAP while in the discussion section they are claiming on average +0.4 t/ha (+3.6 %) higher yield over multiple locations. Please check the same.
2. Line no. 73-73:

In many cases however, the abundant P is fixed by minerals and therefore not fully plant available.

May be written as:

In many cases however, the abundant P is fixed by minerals and therefore not fully available for plants.

3. Line no. 100:

We applied four different starter fertilizers, namely….

Since authors have used three starter fertilizers and one control..So this may be written as:

We applied four treatments i.e. control and three different starter fertilizers…

4. Figure S5 and S6 cited before S4 in text. Please re-arrange figures to cite them sequentially.
